# Prognostic Value of Blood Pressure Variability for Patients With Acute or Subacute Intracerebral Hemorrhage: A Meta-Analysis of Prospective Studies

**DOI:** 10.3389/fneur.2021.606594

**Published:** 2021-03-11

**Authors:** Weidong Liu, Xianbo Zhuang, Liyong Zhang

**Affiliations:** ^1^Department of Neurosurgery, Liaocheng People's Hospital, Liaocheng, China; ^2^Department of Neurology, Liaocheng People's Hospital, Liaocheng, China

**Keywords:** blood pressure variability, intracerebral hemorrhage, prognosis, systolic blood pressure, neurosurgery

## Abstract

The results on the role of systolic blood pressure (SBP) variability in the functional outcome for patients with intracerebral hemorrhage (ICH) have been inconsistent. Hence, this meta-analysis of prospective studies was conducted to assess the association between SBP variability and poor outcomes in patients with acute or subacute ICH. PubMed, Embase, and the Cochrane Library were electronically searched for eligible studies from their inception to July 2020. The role of SBP variability assessed using standard deviation (SD), coefficient of variation (CV), successive variation (SV), average real variability (ARV), and residual standard deviation (RSD) in the risk of poor functional outcomes were assessed using odds ratio (OR) with 95% confidence interval (CI) through the random-effects model. Seven prospective studies involving 5,201 patients with ICH were selected for the final meta-analysis. Increased SBP variability was associated with an increased risk of poor functional outcomes, regardless of its assessment using SD (OR: 1.38; 95% CI: 1.14–1.68; *P* = 0.001), CV (OR: 1.98; 95% CI: 1.13–3.47; *P* = 0.017), SV (OR: 1.30; 95% CI: 1.08–1.58; *P* = 0.006), ARV (OR: 1.13; 95% CI: 1.03–1.24; *P* = 0.010), or RSD (OR: 1.22; 95% CI: 1.00–1.50; *P* = 0.049). Moreover, the role of SBP variability in the risk of poor functional outcomes for patients with ICH was affected by country, study design, mean age, stroke type, outcome definition, and study quality. This study indicated that SBP variability was a predictor of poor outcomes for patients with ICH.

## Introduction

Stroke is the second leading cause of death, accounting for nearly 10% of all deaths worldwide (1). Moreover, it is considered the leading cause of permanent disability and accounts for 5% of the loss of all disability-adjusted life-years (2). Hemorrhagic stroke accounts for nearly 15% of all stroke cases and contributes more to the loss of disability-adjusted life-years compared with ischemic stroke (2, 3). The most common pathophysiological mechanism for acute intracerebral hemorrhage (ICH) was chronic arterial hypertension with the rupture of microscopic pseudoaneurysms in the basal ganglia, thalami, pons, midbrain, and cerebellum (4–6). Furthermore, ICH is characterized by high morbidity, acute onset, and high recurrence, disability, or mortality rate; the mortality rate within 30 days ranged from 30 to 40% (7, 8).

Ambulatory blood pressure (BP) monitoring during a daily cycle was better than traditional clinic BP monitoring to detect BP values for an accurate assessment of BP control and the prognosis of various diseases (9, 10). It could be used to calculate the intraindividual fluctuation in BP levels within 24 h. Studies have already found that BP variability is significantly associated with organ damage and cardiovascular events (11–13). Several systematic reviews and meta-analyses were conducted to assess the role of BP variability in the progression of stroke (14–18). However, the prognostic role of BP variability for patients with acute stroke was less addressed. Manning et al. conducted a systematic review of 18 studies and found that greater systolic BP variability was associated with poor functional outcomes in patients with acute stroke, while only two of the included studies focused on patients with ICH (19). Therefore, this meta-analysis of prospective studies was performed to assess the potential prognostic role of systolic BP variability in patients with ICH.

## Methods and Materials

### Data Sources, Search Strategy, and Selection Criteria

This study was performed and reported following the Meta-analysis of Observational Studies in Epidemiology protocol (20). It investigated the role of systolic BP variability in patients with ICH, with no restriction on publication language and status. The electronic databases of PubMed, Embase, and the Cochrane Library were systematically searched to select eligible studies from their inception up to July 2020. The search terms were as follows: (“Stroke” or “cerebr* vascular disease” or “intracerebr* hemorrhage” or “cerebr* hemorrhage” or “brain hemorrhage”) and (“blood pressure variability” or “BPV”) and (“outcome*” or “prognos*” or “predict*” or “mortality” or “death” or “dependence*” or “disability” or “neurological deterioration” or “functional dependence*”). The reference lists from retrieved studies were also manually searched to identify any new study meeting the inclusion criteria. The subject heading, design, disease status, exposure, and prognostic outcomes of eligible studies were applied to select potentially relevant studies.

The literature search and study selection were conducted by two reviewers independently, and any inconsistency was resolved with the help of an additional reviewer. The inclusion criteria were as follows: (1) patients: all patients with ICH; and (2) exposure: systolic BP variability, including assessment using standard deviation (SD), coefficient of variation (CV), successive variation (SV), average real variability (ARV), and residual standard deviation (RSD); the systolic BP variability parameters were calculated both per 10 mm Hg shift and in quintiles (19); (3) outcomes: poor functional outcome, with the outcome definition given; and (4) study design: prospective studies, including prospective cohort and follow-up randomized controlled trials.

### Data Collection and Quality Assessment

The following data from the studies included were independently collected by two reviewers: first author or study group's name, publication year, country, study design, sample size, mean age, male proportion, stroke phase, systolic BP targets, systolic BP variability, time from stroke onset to recruitment, BP measurement, outcome definition, follow-up duration, covariates adjusted, and effect estimate and its 95% CI. The effect estimate based on crude data and with maximally adjusted covariates was selected if the study reported several multivariable-adjusted effect estimates. The study quality was assessed using the Newcastle-Ottawa Scale (NOS), which was based on selection (four items), comparability (one item), and outcome (three items); the scoring system ranged from 0 to 9 (21). Any study with eight or nine stars was considered to be of high quality. Any inconsistencies between the two reviewers for data collection and quality assessment were settled by discussion until a consensus was reached.

### Statistical Analysis

The prognostic role of systolic BP variability in patients with ICH was assigned as effect estimate [odds ratio (OR), relative risk, and hazard ratio] and 95% confidence interval (CI) in each individual study. After this, the random-effects model was applied to calculate the pooled ORs and 95% CIs for SD, CV, SV, ARV, and RSD of systolic BP variability (22, 23). Heterogeneity across included studies was assessed using *I*^2^ and *Q* statistic, and a *P*-value < 0.10 indicated significant heterogeneity (24, 25). Sensitivity analysis was conducted to assess the stability of pooled conclusions by sequentially excluding individual studies (26). Subgroup analyses for the risk of poor outcomes related to systolic BP variability by SD and CV were conducted based on country, study design, mean age, stroke type, outcome definition, and study quality, and the interaction *P-*value was applied to assess the difference between subgroups (27). Publication biases for poor outcomes related to systolic BP variability assessed using SD, CV, SV, and ARV were evaluated with funnel plots, Egger's test, and Begg's test (28, 29). The inspection level for all pooled analyses were two-sided, and *P* < 0.05 was regarded as statistically significant. Stata software (version 10.0; Stata Corporation, TX, USA) was applied to conduct all statistical analyses.

## Results

### Literature Search

**Figure 1:**
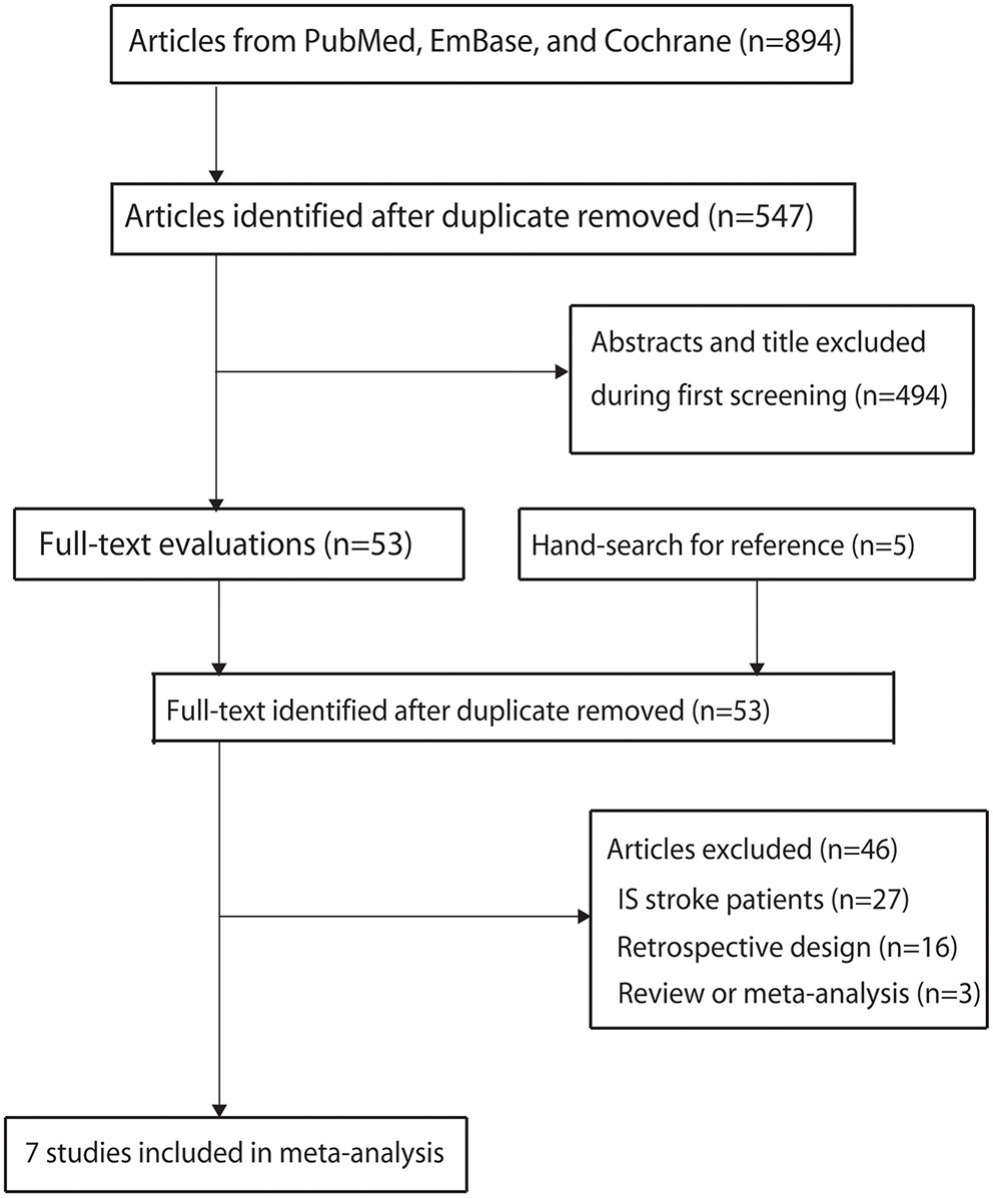
Details regarding the literature search and study selection process.

The details regarding the literature search and study selection are summarized in Figure 1. Overall, 894 records were obtained from initial electronic searches, and 841 were excluded because of duplicate titles and irrelevant studies. A total of 53 studies were retrieved for further full-text evaluations, and 46 studies were excluded due to the following reasons: patients with ischemic stroke (*n* = 27), retrospective design (*n* = 16), and review or meta-analysis (*n* = 3). After detailed evaluations, seven prospective studies were selected for the final quantitative meta-analysis (30–36). No new eligible study was detected by manually searching the reference lists of retrieved studies. The baseline characteristics of the included studies and patients are displayed in Table 1.

**Table 1:** Characteristics of studies and patients included.

**Study**	**Country**	**Study design**	**Sample size**	**Mean age (year)**	**Male (%)**	**Stroke phase**	**Systolic BP targets**	**Systolic BP variability**	**Time from stroke** **onset (h)**	**BP measurement**	**Outcome definition**	**Follow-up (month)**	**Covariates adjusted**	**Study quality**
SAMURAI-ICH 2014 (30)	Japan	Prospective observational analysis	205	65.0	61.0	Acute ICH (initial 24 h)	<160 mm Hg	SD and SV	<3	Casual cuff BP for 24 h (every 15 min during the first 2 h, and every 60 min during the next 22 h)	mRS 4–6	3.0	Sex, age, previous antithrombotic medication, initial systolic BP, initial heart rate, initial NIHSS, onset to treatment time, initial hematoma volume, and serum glucose level at baseline	7
INTERACT2 2014 (31)	International	Observational analysis (RCT data)	2645	63.5	62.1	Hyperacute (first 24 h)/acute ICH (2–7 days)	<140 mm Hg; <180 mm Hg	SD, CV, ARV, and RSD	<6	Casual cuff BP for the first 24 h (every 15 min in the first hour, every 6 h until 24 h)	mRS 3–6	3.0	Age, sex, randomized group, region, hematoma volume at baseline, high scores on the NIHSS, and mean systolic BP during each period	9
ATACH-2 2018 (32)	USA	Observational analysis (RCT data)	913	62.1	61.7	Acute (2–24 h)/subacute ICH (2–7 days)	<140 mm Hg; <180 mm Hg	SD, CV, ARV, SV, and RSD	<48	Casual cuff BP for the acute (highest and lowest SBP/h) and subacute period (the two highest and lowest SBP readings separated by 1 h for days 2, 3, and 7)	mRS 3–6	3.0	Age, baseline NIHSS, premorbid antihypertensive medication, intraventricular hemorrhage, and laterality of ICH	8
Zhang 2018 (33)	China	Prospective observational analysis	131	60.2	60.3	Acute ICH (first 24 h)	NA	SD and CV	<6	Casual cuff BP every 15 min from admission to 1 h, once every 30 min from 1 to 6 h, and once every hour from 6 to 24 h	mRS 2–6	3.0	Hypertension, DM, metabolic syndrome, smoking, and stroke history	6
Jeon 2018 (34)	Korea	Prospective observational analysis	104	63.0	57.7	Acute ICH (within 7 days)	<140 mm Hg	SD, CV, and ARV	<6	BP was monitored every 15 min during the first 2 h and hourly until the follow-up computed tomography scan and during the entire admission period in the intensive care units	mRS 3–6	3.0	Female, initial hematoma volume, hypertension, LDL cholesterol, mean BP, and range	6
FAST-MAG 2018 (35)	USA	Observational analysis (RCT data)	386	65.5	66.6	Hyperacute (first 4–6 h)/acute ICH (first 24–26 h)	NA	SD, CV, and SV	<2	Casual cuff at 11 time points (the first BP measurement was performed at the time of first paramedic patient assessment; the second BP assessment was performed on ED arrival, before maintenance study medication infusion, 15 min and 1 hour after the start of the maintenance infusion, and 4, 8, 12,16, 20, and 24 h after ED arrival) during the first 24 h	mRS 3–6	3.0	Baseline stroke severity, age, presence of pre-stroke disability, geographic region of enrolling ambulance, sex, BMI, CAD, alcohol habit, blood urea nitrogen, eGFR, hemoglobin, and mean systolic BP	9
HeadPoST 2019 (36)	International	Observational analysis (RCT data)	817	68.1	60.8	Acute ICH (first 24 h)	NA	CV	<24	Casual cuff at 4-hourly intervals during the first 24 h	mRS 3–6	3.0	Country, pre-stroke mRS score, sex, baseline NIHSS score, history of heart disease, stroke, DM, or hypertension, and prior antiplatelet therapy	8

*ARV, Average real variability; BMI: body mass index; BP, blood pressure; CAD: coronary artery disease; CV, coefficient of variation; DM: diabetes mellitus; eGFR: estimated glomerular filtration rate; ICH: intracerebral hemorrhage; mRS, modified Rankin Scale; NA: not available; NIHSS, National Institutes of Health Stroke Scale; RSD, residual standard deviation; SD, standard deviation; SV, successive variation*.

### Study Characteristics

Of seven included studies, three were prospective observational studies, and the remaining four were a follow-up of randomized controlled trials. A total of 5,201 patients with ICH were recruited, and 104–2,645 patients were included in each study. Two studies were conducted in multiple countries, two were conducted in the USA, and the remaining three studies were conducted in Asia (China, Japan, and Korea). Two studies included patients in the hyperacute stage, seven included patients in the acute stage, and one included patients in the subacute stage. The study quality was assessed using NOS; two studies had nine stars, two had eight stars, one had seven stars, and the remaining two had six stars.

### Meta-Analysis

**Figure 2:**
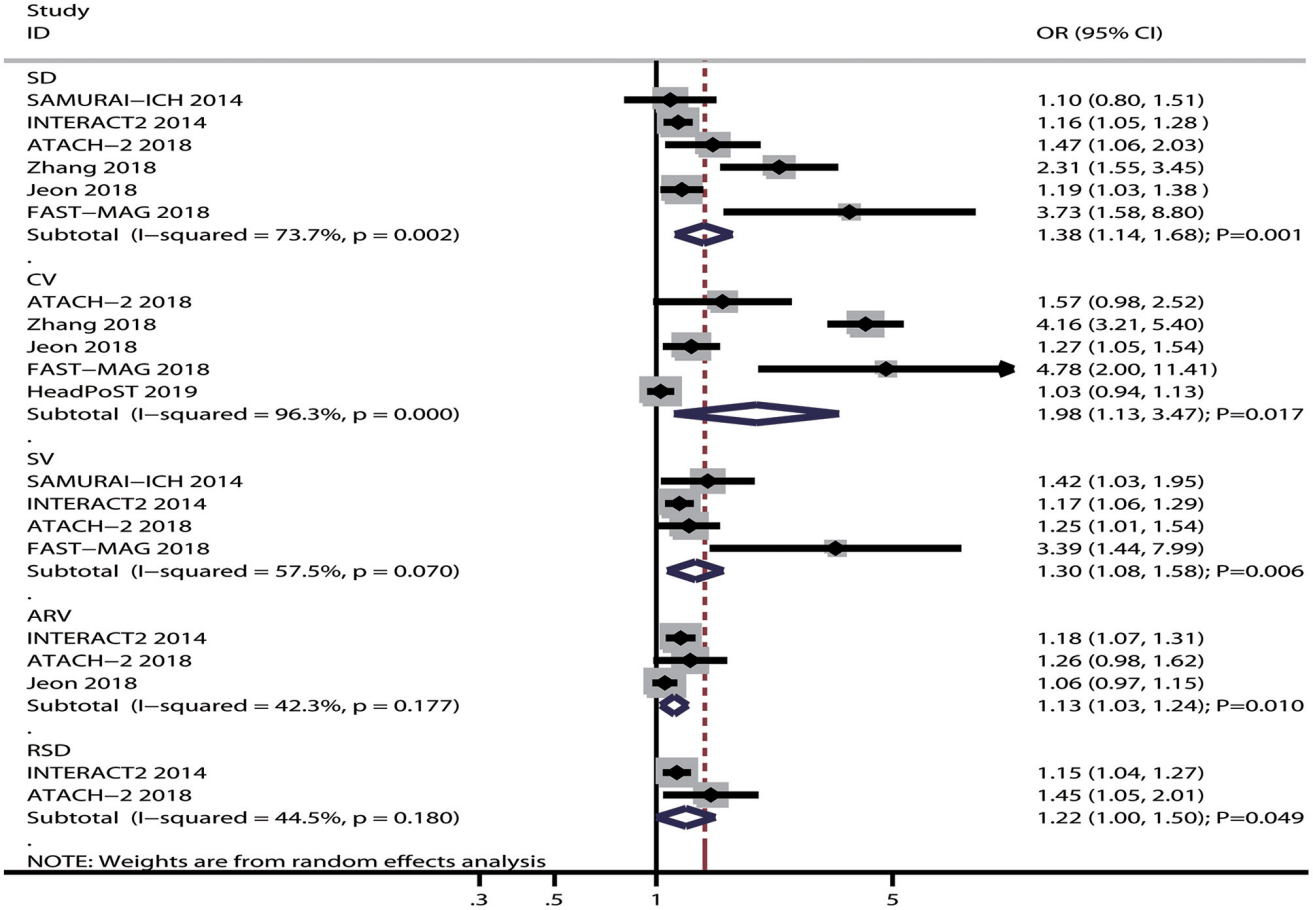
Forest plot of the association between systolic BP variability and poor functional outcome.

**Figure 3:**
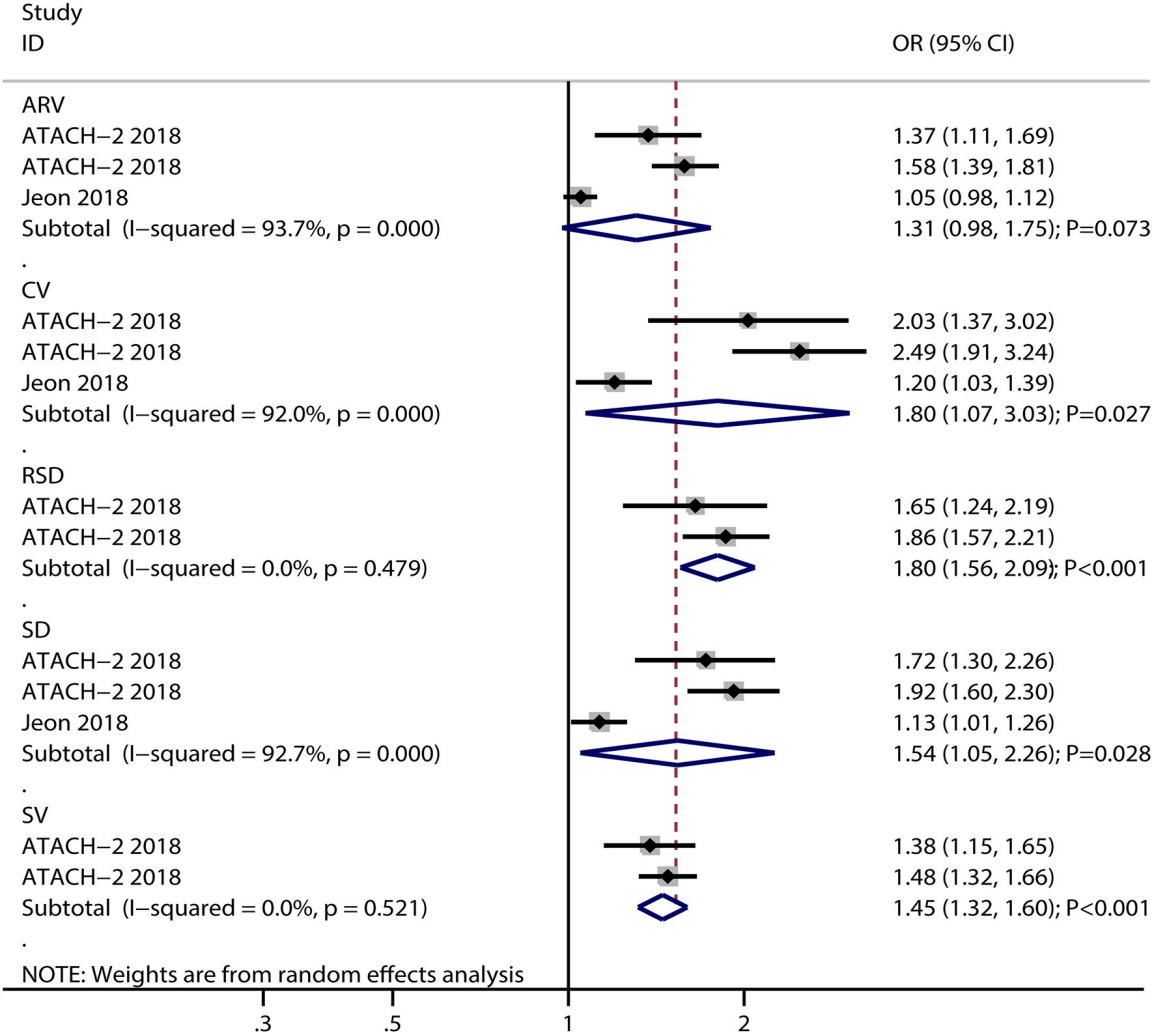
Forest plot for the association of systolic BP variability with poor functional outcome based on crude data.

The breakdown for the number of studies available for systolic BP variability assessed using SD, CV, SV, ARV, and RSD was 6, 5, 4, 3, and 2 studies, respectively (Figure 2). Systolic BP variability assessed, using SD (OR: 1.38; 95% CI: 1.14–1.68; *P* = 0.001), CV (OR: 1.98; 95% CI: 1.13–3.47; *P* = 0.017), SV (OR: 1.30; 95% CI: 1.08–1.58; *P* = 0.006), ARV (OR: 1.13; 95% CI: 1.03–1.24; *P* = 0.010), or RSD (OR: 1.22; 95% CI: 1.00–1.50; *P* = 0.049), was associated with an increased risk of poor functional outcomes for patients with ICH. Moreover, significant heterogeneity was noted across included studies for systolic BP variability assessed using SD (*I*^2^ = 73.7%; *P* = 0.002), CV (*I*^2^ = 96.3%; *P* < 0.001), and SV (*I*^2^ = 57.5%; *P* = 0.070), while moderate heterogeneity was observed across included studies for systolic BP variability assessed using ARV (*I*^2^ = 42.3%; *P* = 0.177) and RSD (*I*^2^ = 44.5%; *P* = 0.180). While pooling crude data, systolic BP variability assessed using SD (OR: 1.54; 95% CI: 1.05–2.26; *P* = 0.028), CV (OR: 1.80; 95% CI: 1.07–3.03; *P* = 0.027), SV (OR: 1.45; 95% CI: 1.32–1.60; *P* < 0.001), and RSD (OR: 1.80; 95% CI: 1.56–2.09; *P* < 0.001) was found to be associated with an increased risk of poor functional outcomes, whereas ARV was not associated with the risk of poor functional outcomes (OR: 1.31; 95% CI: 0.98–1.75; *P* = 0.073) (Figure 3). A significant heterogeneity was observed for systolic BP variability assessed using SD (*I*^2^ = 92.7%; *P* < 0.001), CV (*I*^2^ = 92.0%; *P* < 0.001), and ARV (*I*^2^ = 93.7%; *P* < 0.001), while no evidence of heterogeneity was found for systolic BP variability assessed using SV (*I*^2^ = 0.0%; *P* = 0.521) and RSD (*I*^2^ = 0.0%; *P* = 0.479).

### Sensitivity Analysis

The results of sensitivity analyses for the prognostic role of systolic BP variability in the risk of poor functional outcomes are presented in Table 2. First, the prognostic role of systolic BP variability by SD was robust and not altered by sequentially excluding individual studies; the pooled OR ranged from 1.24 to 1.54, and the heterogeneity across included studies remained high. Second, the pooled conclusion for the risk of poor functional outcomes related to systolic BP variability by CV varied, and the pooled OR ranged from 1.40 to 2.42. Moreover, the heterogeneity across the studies included remained high and was not fully explained by sensitivity analysis. Third, the prognostic role of systolic BP variability by SV in the risk of poor functional outcomes was stable, with no evidence of heterogeneity among the studies included after excluding the FAST-MAG study (35). Fourth, the pooled conclusion on the role of systolic BP variability by ARV varied, with no evidence of heterogeneity after excluding the study conducted by Jeon et al. (34). Finally, the pooled conclusion on the role of systolic BP variability by RSD in the risk of poor functional outcomes was robust, and the pooled OR ranged from 1.15 to 1.45.

**Table 2:** Sensitivity analysis for the risk of poor outcomes.

**Blood pressure variability**	**Study omitted**	**OR and 95% CI**	***P*-value**	**Heterogeneity (%)**	***P*-value for heterogeneity**
SD	SAMURAI-ICH 2014	1.46 (1.16–1.84)	0.001	78.5	0.001
	INTERACT2 2014	1.54 (1.14–2.08)	0.005	76.0	0.002
	ATACH-2 2018	1.38 (1.10–1.72)	0.005	77.3	0.001
	Zhang 2018	1.24 (1.07–1.44)	0.005	55.2	0.063
	Jeon 2018	1.53 (1.13–2.07)	0.006	78.8	0.001
	FAST-MAG 2018	1.30 (1.10–1.54)	0.002	67.8	0.014
CV	ATACH-2 2018	2.10 (1.09–4.05)	0.026	97.2	< 0.001
	Zhang 2018	1.40 (1.02–1.93)	0.038	82.5	0.001
	Jeon 2018	2.30 (0.95–5.59)	0.066	97.2	< 0.001
	FAST-MAG 2018	1.70 (0.94–3.08)	0.079	97.0	< 0.001
	HeadPoST 2019	2.42 (1.15–5.07)	0.020	94.7	< 0.001
SV	SAMURAI-ICH 2014	1.30 (1.02–1.65)	0.037	66.8	0.049
	INTERACT2 2014	1.49 (1.06–2.09)	0.022	60.3	0.081
	ATACH-2 2018	1.44 (1.01–2.06)	0.044	71.2	0.031
	FAST-MAG 2018	1.20 (1.10–1.31)	< 0.001	0.0	0.480
ARV	INTERACT2 2014	1.11 (0.96–1.28)	0.174	38.7	0.202
	ATACH-2 2018	1.11 (1.00–1.24)	0.043	60.4	0.112
	Jeon 2018	1.19 (1.08–1.31)	< 0.001	0.0	0.635
RSD	INTERACT2 2014	1.45 (1.05–2.01)	0.025	–	–
	ATACH-2 2018	1.15 (1.04–1.27)	0.004	–	–

### Subgroup Analysis

Subgroup analyses of the role of systolic BP variability by SD and CV in the risk of poor functional outcomes were conducted, and the results are presented in Table 3. Systolic BP variability by SD was associated with an increased risk of poor functional outcomes in pooled studies conducted in multiple countries (OR: 1.16; 95% CI: 1.05–1.28; *P* = 0.003), studies designed as the follow-up of randomized controlled trials (OR: 1.51; 95% CI: 1.01–2.27; *P* = 0.045), studies with the mean age of patients <65.0 years (OR: 1.35; 95% CI: 1.11–1.65; *P* = 0.003), studies on patients with acute ICH (OR: 1.89; 95% CI: 1.23–2.91; *P* = 0.004) or subacute ICH (OR: 1.56; 95% CI: 1.26–1.93; *P* < 0.001), studies using mRS 2–6 (OR: 2.31; 95% CI: 1.55–3.45; *P* < 0.001) or 3–6 (OR: 1.28; 95% CI: 1.07–1.54; *P* = 0.007) defined as poor functional outcome, and high-quality studies (OR: 1.51; 95% CI: 1.01–2.27; *P* = 0.045). Moreover, systolic BP variability by CV was associated with an increased risk of poor functional outcomes in patients with hyperacute ICH (OR: 4.78; 95% CI: 2.00–11.41; *P* < 0.001) and acute ICH (OR: 2.26; 95% CI: 1.31–3.91; *P* = 0.003), or subacute ICH (OR: 1.88; 95% CI: 1.38–2.56; *P* < 0.001) and also in studies using mRS 2–6 (OR: 4.16; 95% CI: 3.21–5.40; *P* < 0.001) or 3–6 (OR: 1.40; 95% CI: 1.02–1.93; *P* = 0.038) defined as poor functional outcome.

**Table 3:** Subgroup analyses for the risk of poor outcome.

**BPV**	**Factors**	**Group**	**OR and 95% CI**	***P*-value**	**Heterogeneity (%)**	***P*-value for heterogeneity**	***P-*value between subgroups**
SD	Country	International	1.16 (1.05–1.28)	0.003	–	–	0.082
		USA	2.14 (0.88–5.25)	0.095	74.7	0.047	
		Asia	1.40 (0.97–2.01)	0.070	80.1	0.007	
	Study design	Prospective observational	1.40 (0.97–2.01)	0.070	80.1	0.007	0.582
		RCT data	1.51 (1.01–2.27)	0.045	76.9	0.013	
	Mean age (year)	≥65.0	1.89 (0.58–6.22)	0.293	85.4	0.009	0.776
		<65.0	1.35 (1.11–1.65)	0.003	75.1	0.007	
	Stroke phase	Hyperacute	1.92 (0.62–5.96)	0.260	85.7	0.008	0.004
		Acute	1.89 (1.23–2.91)	0.004	95.9	< 0.001	
		Subacute	1.56 (1.26–1.93)	< 0.001	–	–	
	Outcome definition	mRS 2–6	2.31 (1.55–3.45)	< 0.001	–	–	0.006
		mRS 3–6	1.28 (1.07–1.54)	0.007	65.4	0.034	
		mRS 4–6	1.10 (0.80–1.51)	0.552	–	–	
	Study quality	High	1.51 (1.01–2.27)	0.045	76.9	0.013	0.582
		Low to moderate	1.40 (0.97–2.01)	0.070	80.1	0.007	
CV	Country	International	1.03 (0.94–1.13)	0.529	–	–	< 0.001
		USA	2.57 (0.87–7.61)	0.087	79.4	0.028	
		Asia	2.29 (0.72–7.33)	0.162	98.0	< 0.001	
	Study design	Prospective observational	2.29 (0.72–7.33)	0.162	98.0	< 0.001	< 0.001
		RCT data	1.74 (0.88–3.43)	0.111	86.2	0.001	
	Mean age (year)	≥65.0	2.08 (0.47–9.32)	0.338	91.5	0.001	< 0.001
		<65.0	2.03 (0.88–4.68)	0.096	96.2	< 0.001	
	Stroke phase	Hyperacute	4.78 (2.00–11.41)	< 0.001	–	–	0.001
		Acute	2.26 (1.31–3.91)	0.003	96.3	< 0.001	
		Subacute	1.88 (1.38–2.56)	< 0.001	–	–	
	Outcome definition	mRS 2–6	4.16 (3.21–5.40)	< 0.001	–	–	< 0.001
		mRS 3–6	1.40 (1.02–1.93)	0.038	82.5	0.001	
	Study quality	High	1.74 (0.88–3.43)	0.111	86.2	0.001	< 0.001
		Low to moderate	2.29 (0.72–7.33)	0.162	98.0	< 0.001	

### Publication Bias

**Figure 4:**
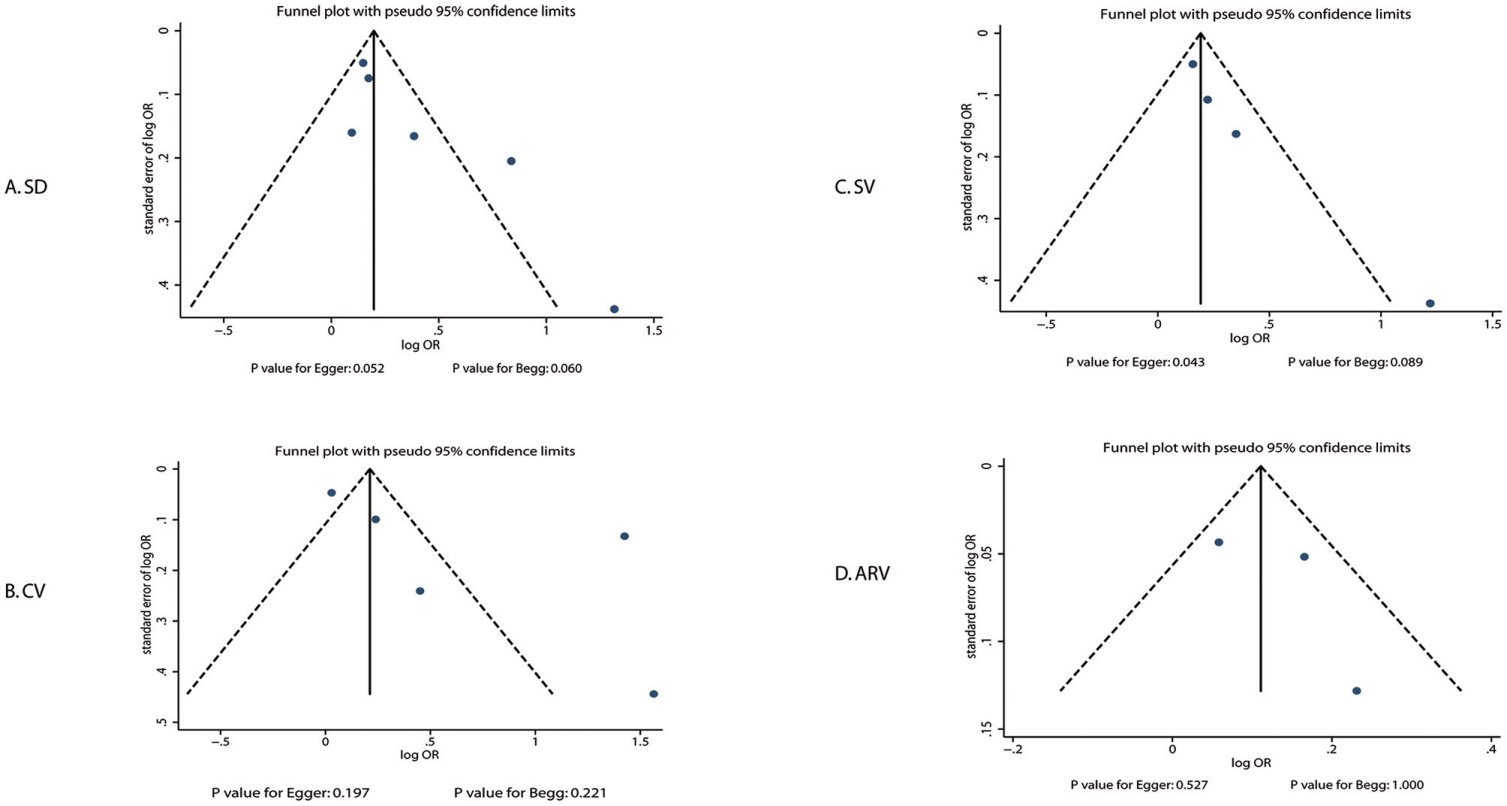
Publication biases for the association between systolic BP variability and poor functional outcome.

A review of the funnel plots could not rule out the potential for publication bias for the prognostic role of systolic BP variability by SD, CV, SV, and ARV in the risk of poor functional outcomes (Figure 4). No significant publication biases were observed for the role of systolic BP variability by CV (*P*-value for Egger: 0.197; *P*-value for Begg: 0.221) and ARV (*P*-value for Egger: 0.527; *P*-value for Begg: 1.000), but potential publication biases were noted for the role of systolic BP variability by SD (*P*-value for Egger: 0.052; *P*-value for Begg: 0.060) and SV (*P*-value for Egger: 0.043; *P*-value for Begg: 0.089). The conclusions were not changed after adjusting for publication bias using the trim-and-fill method (37).

## Discussion

This systematic review and meta-analysis was based on prospective studies and explored all possible correlations between systolic BP variability and the risk of poor functional outcomes. This comprehensive quantitative analysis involved 5,201 patients with ICH from seven prospective studies across a wide range of characteristics, studies, and patients. The study showed that increased systolic BP variability was associated with an increased risk of poor functional outcomes for patients with ICH. The analysis of crude data indicated that most parameters of systolic BP variability (SD, CV, SV, and RSD) could cause excess risk of poor functional outcomes, while ARV did not affect the risk of poor functional outcomes. Moreover, subgroup analyses suggested that the prognostic value of systolic BP variability in the risk of poor functional outcomes was more evident in studies conducted in multiple countries, studies designed as a follow-up of randomized controlled trials, studies with a mean patient age of <65.0 years, studies involving patients with acute ICH or subacute ICH, studies using mRS 2–6 or 3–6 defined as poor functional outcomes and high-quality studies.

Several systematic reviews and meta-analyses have already illustrated the potential role of BP variability in the progression and prognosis of stroke (14–19). However, most studies focused on a general population and assessed the role of systolic BP variability in the primary prevention of the risk of subsequent stroke (14–18). Few studies included investigated the role of systolic BP variability for patients with ICH. Moullaali et al. performed a pooled analysis based on individual participant data and found that achieving early and stable systolic BP was associated with better outcomes for patients with ICH. The present analysis was based on the participants from INTERACT2 and ATACH-II trials (31, 32); additional trials were not included (38). The analysis involved all available prospective studies. Also, the BP measurement and the definition of stroke phase differed across included studies, thus playing an important role in significant heterogeneity among the included studies.

This study found that large systolic BP variability was associated with an increased risk of poor functional outcomes in patients with ICH. The potential reason for this could be the variability in BP when assessed using various indexes. The stability and reliability of the predictive model should be further explored using various indexes for assessing systolic BP variability. Studies have already demonstrated that higher BP is associated with an increased risk of early deterioration, hematoma growth, and worse final functional outcome; lowering BP for patients with ICH should be recommended to prevent hemorrhage expansion in clinical practice (39–41). However, the results regarding the aggressive lowering of BP for patients with ICH were inconsistent (42, 43). Although BP variability was independently associated with poor functional outcomes after ICH, this association depended on the time window (31, 44). Therefore, further studies should be conducted to explore the role of BP variability in measuring the time window for the prognosis of ICH.

Subgroup analyses found that the prognostic role of systolic BP variability in the risk of poor functional outcomes in patients with ICH was affected by country, study design, mean age, stroke type, outcome definition, and study quality. The potential reasons for this were as follows: (1) the study conducted in multiple countries included a large number of patients, and the results were stable; (2) data from the follow-up of randomized controlled trials were better than prospective cohort data because the confounders were well-controlled; (3) younger patients could better restore ability, and systolic BP variability was more stable in young patients than in elderly patients; 4) systolic BP variability for patients with ICH in various stages differed, and greater systolic BP variability for patients in super acute and acute stages might be associated with worse prognosis; (5) the definition of poor functional outcome could affect the events and was significantly related to the power to detect potential associations; and (6) the reliability of results significantly correlated with the study quality.

In the planning stage, the role of BP variability in the prognosis of ICH should be fully evaluated; however, only one trial reported the association between BP variability and hematoma growth (30). The results of this study confirmed the important feature for the monitoring of BP after ICH, especially within 7 days. Moreover, the strength for the relationship between BP variability and prognosis of ICH were further explored in subgroup analyses. Further, the high-risk population should be monitored in clinical practice.

This study had several limitations. First, the included patients were in different stages, which could have affected the prognosis of ICH. Second, the heterogeneity across the studies included was not fully explained using sensitivity and subgroup analyses, which might be related to various outcome definitions, systolic BP targets, and background therapies. Third, the background therapies for managing hypertension differed across included studies, which might have influenced BP variability and outcomes. Fourth, various covariates were adjusted among the studies included, and these covariates might have affected the prognosis of ICH. Fifth, all of the studies included investigated the role of systolic BP variability in ICH in hyperacute, acute, and subacute stages; no study focused on patients in other stages. Sixth, the analysis was based on pooled data, and the detailed analyses were restricted. Finally, this study was based on published studies, and hence publication bias was inevitable.

In conclusion, this study showed that large systolic BP variability was associated with an increased risk of poor functional outcomes in patients with ICH. Moreover, the prognostic role of systolic BP variability could be affected by country, study design, mean age, stroke type, outcome definition, and study quality. These conclusions suggested that increased intraindividual fluctuation of systolic BP should be cautiously managed to improve the prognosis of ICH. Further large-scale prospective studies should be conducted to assess the stability of the predictive model for the systolic BP variability in patients with ICH.

## Data Availability Statement

The original contributions presented in the study are included in the article/supplementary material, further inquiries can be directed to the corresponding author/s.

## Author Contributions

WL and LZ carried out the experiments, participated in collecting data, and drafted the manuscript. XZ performed statistical analysis and participated in its design. All authors read and approved the final manuscript.

## Conflict of Interest

The authors declare that the research was conducted in the absence of any commercial or financial relationships that could be construed as a potential conflict of interest.
